# Continuous Support Promotes Obstetric Labor Progress and Vaginal Delivery in Primiparous Women – A Randomized Controlled Study

**DOI:** 10.3389/fpsyg.2021.582823

**Published:** 2021-02-12

**Authors:** Ylva Vladic Stjernholm, Paula da Silva Charvalho, Olga Bergdahl, Tomislav Vladic, Maria Petersson

**Affiliations:** ^1^Department of Women's and Children's Health Obstetric Unit, Karolinska University Hospital and Karolinska Institutet, Stockholm, Sweden; ^2^Department of Women's and Children's Health, Karolinska University Hospital and Karolinska Institutet, Stockholm, Sweden; ^3^Department of Endocrinology, Karolinska University Hospital and Karolinska Institutet, Stockholm, Sweden

**Keywords:** birth, cesarean and vaginal deliveries, cortisol, continuous support, labor & delivery, oxytocin, stress

## Abstract

**Background:** Obstetric labor and childbirth are mostly regarded as a physiological process, whereas social, cultural, psychological and transcendental aspects have received less attention. Labor support has been suggested to promote labor progress. The aim of this study was to investigate whether continuous labor support by a midwife promotes labor progress and vaginal delivery.

**Material and Methods:** A randomized controlled study at a university hospital in Sweden in 2015–17. Primiparous women with singleton pregnancy and spontaneous labor onset were randomized to continuous support (*n* = 30) or standard care (*n* = 29) during delivery. The primary outcome was the duration of active labor. Secondary outcomes were delivery mode, women's need of labor analgesia and satisfaction with delivery, maternal cortisol levels, and neonatal morbidity.

**Results:** Continuous support was followed by shorter active labor 11.0 ± 5.7 h compared to 13.7 ± 3.9 h with standard care (*p* = 0.001). Women in the continuous support group tended to have lower cortisol levels and low cortisol during the first (*p* = 0.02) and second (*p* = 0.04) stages of labor were correlated with shorter active labor. Continuous support was followed by spontaneous delivery in 73%, instrumental delivery in 24% and emergency cesarean section in 3% in contrast to standard care which was followed by spontaneous delivery in 62%, instrumental delivery in 24% and cesarean in 14% (*p* = 0.19). The continuous support group received combined analgesic methods more often (*p* = 0.04). Women's satisfaction with delivery and neonatal morbidity were comparable.

**Conclusion:** Continuous labor support was followed by shorter active labor compared to standard care. Women with continuous support had a high rate of vaginal delivery and tended to have lower cortisol levels during all stages of active labor reflecting a lower stress level. Low cortisol was correlated to shorter active labor. Based on these results, we recommend continuous labor support for all primiparous women during active labor.

## Introduction

Obstetric labor and childbirth are mostly regarded as a physiological process, whereas social, cultural, psychological, and transcendental aspects have received less attention (Kennell et al., 1991; Bohren et al., 2017; World Health Organization, 2018; Uvnas-Moberg et al., 2019; Olza et al., 2020). Women's experiences of maternity care and childbirth are worldwide public health care issues and critical for high-quality obstetric care according to the World Health Organization (WHO) (World Health Organization, 2018). Psychological and physical labor support has been suggested to promote labor progress and to reduce repeated unnecessary interventions, possibly by reducing maternal anxiety (Kennell et al., 1991; Bohren et al., 2017; World Health Organization, 2018; Uvnas-Moberg et al., 2019; Olza et al., 2020). Continuous support during delivery includes actions for the promotion of psychological well-being such as presence, encouragement, reassurance and consolation and actions to promote physical well-being such as touch and help for the laboring woman to communicate her opinions and needs to the medical staff (Kennell et al., 1991; Bohren et al., 2017; World Health Organization, 2018; Uvnas-Moberg et al., 2019; Olza et al., 2020).

Components of labor support such as human reassurance and touch are known to facilitate release of the peptide hormone oxytocin, which stimulates uterine myometrial contractions and labor progress. In addition, oxytocin induces several effects that counteract stress including calm, joy and empowerment, as well as reduction of fear, anxiety and pain and decreased cortisol levels (Uvnas-Moberg et al., 2019; Olza et al., 2020). Exogenous oxytocin infusion is used for labor augmentation in standard clinical practice (Zhang et al., 2010). In contrast, a high extent of emotional stress, anxiety, fear and pain are associated with prolonged labor (Lederman et al., 1985; Kono et al., 1987; Stjernholm et al., 2016; Hishikawa et al., 2019). The stress hormones catecholamines and cortisol increase in laboring women and high levels of catecholamines are correlated to reduced uterine activity and prolonged labor (Lederman et al., 1985; Kono et al., 1987; Stjernholm et al., 2016; Hishikawa et al., 2019). Maternal cortisol is higher during labor in primipaous women than in parous women, indicating a higher stress level among first time mothers (Kono et al., 1987). The oxytocin system and stress hormone systems act independently during labor, but may also inhibit the effect of each other (Bohren et al., 2017; Uvnas-Moberg et al., 2019).

Further knowledge about whether continuous support promotes obstetric labor progress and spontaneous delivery is of high importance in order to improve maternal and infant health and to reduce unnecessary interventions during childbirth. Most instrumental deliveries and emergency cesareans (CS) are performed because of prolonged labor, and these interventions increase the risks of complications such as obstetric bleeding, infection, perineal lacerations and a negative childbirth experience (Zhang et al., 2010; Simic et al., 2017; da Silva Charvalho et al., 2019). The aim of this study was to investigate whether continuous labor support by a midwife promotes labor progress among primiparous women.

## Materials and Methods

This study was conducted at the Obstetric Unit, Department of Women's and Children's Health, Karolinska University Hospital Solna, Stockholm, Sweden between April 1, 2015 and December 31, 2017. Ethics approval was obtained from the Regional Ethics Board for Medical Sciences in Stockholm October 9, 2013, Ref No. 2013/1079–31/3.

### Study Participants

Study participants were healthy, primiparous women with uncomplicated singleton pregnancy and spontaneous labor onset at a normal gestational length between 37 and 41 weeks. Exclusion criteria were maternal age <18 years, multiple pregnancy, induced delivery, intercurrent diseases, pregnancy complications, intrauterine growth restriction, fetal anomalies, or signs of fetal distress according to fetal heart monitoring with cardiotocography (CTG) or ultrasonography.

Eligible women were asked for participation by a midwife or physician at admission to the Obstetric Unit during the early first stage of labor at cervical dilatation of ≤4 cm. All women were informed that they were about to be part of a study and they were included after individual oral and written consent. The randomization process was blinded using a computerized randomization system with closed envelopes. The group allocation was blinded to the participants but not to the obstetric team. Active labor was the interval between cervical dilatation of 4 cm and childbirth in 2015–17. The first stage of labor (opening stage) was the duration between cervical dilatation of 4 cm and complete cervical dilatation. The second stage was the interval between complete cervical dilatation and childbirth. The third stage was the interval between childbirth and delivery of the placenta +2 h according to the WHO. The intervention continuous labor support (*n* = 30) was continuous support by a midwife or nurse in the delivery room during the first stage of active labor. The midwives and nurses did not receive any special training before the study. Women with standard care (*n* = 29) received intermittent support by a midwife or nurse during the first stage of active labor in periods of 5–15 min according to standard clinical routines. All women in both groups received continuous support by a midwife and nurse during the second stage according to standard clinical routines. The woman was seen by a physician in case of prolonged labor, non-reassuring fetal heart monitoring or need of labor analgesia, according to standard clinical routines.

Maternal data such as age, body mass index (BMI), gestational age at delivery, delivery mode, obstetric bleeding, need of labor analgesia and satisfaction with delivery were monitored. Progress of labor was assessed by clinical examination of a midwife or an obstetrician and was recorded in the electronic obstetric partogram (Obstetrix® Cerner AB, Stockholm, Sweden). The definition of prolonged labor (labor arrest) was failure to progress for more than 3–4 h during the first stage of labor or more than 2–3 h during the second stage. Maternal oxygen saturation and pulse was continuously monitored using a pulse oximeter, and intermittent manual non-invasive blood pressure measurement was recorded during all deliveries. All women had continuous fetal heart monitoring with CTG during active labor. Signs of fetal asphyxia were a pathological CTG registration according to standardized criteria by The International Federation of Gynecology and Obstetrics (FIGO) or a pathological scalp-lactate blood sample >4.8 mmol/L.

Women's satisfaction with delivery was scored according to standard clinical routines before discharge from the hospital with a standard visual analog scale (VAS) 0–100 mm, where 0 = worst imaginable and 100 = best imaginable experience. Maternal peripheral venous samples for cortisol analyses were obtained during the first stage of labor at 4–6 cm cervical dilatation, during the second stage after complete cervical dilatation, and during the third stage within 2 h after delivery of the placenta. Analyses of serum cortisol were performed at the Department of Clinical Chemistry, Karolinska University Hospital, using electrochemical luminescence, Modular E, Roche Diagnostics, Mannheim, Germany.

Neonatal data such as birth weight (BW), signs of neonatal asphyxia defined as an Apgar score <7 at 5 min or an umbilical artery base excess [BE(a)] < -10 mmol/L, as well as other signs of neonatal distress necessitating admission to a Neonatal Intensive Care Unit (NICU) were monitored.

### Statistical Analysis

Based on clinical observations and previous reports we hypothesized that continuous labor support by a midwife would decrease the duration of active labor by 30% compared to standard care (Bohren et al., 2017). According to a power analysis, a sample size of *n* = 29 in each group would be needed when aiming at a significance of 5% and a power of 80% (Pocock, 1984). Continuous data were analyzed using Mann Whitney U-test and General Linear Model when appropriate and categorical data with One way ANOVA. Continuous data were presented as means ± SD and categorical data as numbers and percentages. A two-tailed *p*-value < 0.05 was considered significant.

## Results

Approximately 3,500 deliveries out of the 112 000 deliveries per year in Sweden take place at the Karolinska University Hospital Solna. During the study period *n* = 9,991 women with singleton pregnancy and *n* = 205 women with multiple pregnancy gave birth at the hospital. Approximately half of the women were primiparous.

The primary outcome was the duration of active labor. Secondary outcomes were delivery mode, women's need of labor analgesia and satisfaction with delivery, maternal cortisol levels, and neonatal morbidity.

Maternal data are shown in Table 1. Demographic data were comparable between the groups. The duration of active labor (mean ± SD) was shorter 11.0 ± 5.7 h among women with continuous support compared to 13.7 ± 3.9 h in women with standard care (*p* = 0.001). The 95% confidence interval (CI) was 158.5–255.7 min in continuous support group as compared to 321.1–515.8 min in the standard care group. The first stage of active labor was 9.7 ± 4.3 h with continuous support compared to 11.1 ± 3.8 h with standard care (*p* = 0.35), and the duration of the second stage was 2.3 ± 1.4 h with continuous support and 2.6 ± 1.5 h with standard care (*p* = 0.52).

**Table 1:** Maternal data.

**Variable**	**Continuous support** ***n* = 30**	**Standard care** ***n* = 29**	***p*-value**
Age, years (mean ± SD)	27 ± 4.4	30 ± 5.7	0.20^a^
BMI, kg/m^2^ (mean ± SD)	24 ± 3.7	23 ± 3.7	0.18^a^
Gest age, weeks ± days (mean)	40 ± 3	40 ± 1	0.56^a^
Labor analgesia			
– EDA, *n* (%) – EDA doses, *n* (mean ± SD)	23 (77) 3.7 ± 3.2	26 (90) 5.1 ± 3.9	0.42^b^ 0.19^a^
– Nitrous oxide, *n* (%)	18 (60)	22 (76)	0.42^b^
– Combined methods, *n* (%)	9 (30)	4 (14)	0.04^b^
Labor augmentation with oxytocin, *n* (%)	23 (79)	26 (90)	0.48^a^
Bleeding, mL (mean ± SD)	449 ± 310	426 ± 215	0.66^a^
Active labor, hours (mean ± SD)	11.0 ± 5.7	13.7 ± 3.9	0.001^a^
– First stage, hours (mean ± SD)	9.7 ± 4.3	11.1 ± 3.8	0.35^a^
– Second stage, hours (mean ± SD)	2.3 ± 1.4	2.6 ± 1.5	0.52^a^
Delivery mode, *n* (%)			0.19^b^
– Spontaneous	22 (73)	18 (62)	
– Instrumental	7 (23)	7 (24)	
– Cesarean	1 (3)	4 (14)	
Satisfaction with delivery, *n* (%) VAS, mm (mean ± SD)	28 (93) 7.7 ± 2.0	27 (93) 7.8 ± 2.1	0.61^a^

a Statistical methods Mann Whitney U-test and General Linear Model when appropriate and

b *One Way ANOVA*.

*BMI, body mass index; EDA, epidural analgesia; Gest age, gestational age at childbirth; Nitrous oxide, inhalation of a mixture of oxygen/nitrous oxide (50/50%); SD, standard deviation; VAS, visual analog scale*.

The delivery mode with continuous support was spontaneous delivery in 73% (*n* = 22/30), instrumental delivery in 23% (*n* = 7/30), and emergency CS in 3% (*n* = 1/30), whereas standard care was followed by spontaneous delivery in 62% (*n* = 18/29), instrumental delivery in 24% (*n* = 7/29), and CS in 14% (*n* = 4/29) (*p* = 0.19).

Women in the continuous support group used epidural analgesia (EDA) in 77% (*n* = 23/30) compared with 90% (*n* = 26/29) in the standard care group (*p* = 0.42), and they used 3.7 ± 3.2 doses of EDA compared to 5.1 ± 3.9 (*p* = 0.19). Women with continuous support used inhalation of a mixture of oxygen and nitrous oxide (50:50%) in 60% (*n* = 18/30) compared to 90% (*n* = 26/29) (*p* = 0.42). The continuous support group received combined modes of labor analgesia such as inhalation of a mixture of nitrous oxide and oxygen (50/50%), acupuncture and /or pudendus blockage more often in 30% (*n* = 9/30) compared to 14% (*n* = 4/29) the standard care group (*p* = 0.04). Linear regression analysis of duration of active labor in dependence of combined modes of labor analgesia revealed a significant negative correlation −0.26 (*p* = 0.03).

Presence of a midwife or nurse in the labor room was documented for 4.9 ± 3.6 h (50%) and 1.9 ± 1.2 h (83%) of the time during the first and second stages of active labor in the continuous support group. The corresponding rates in the standard care group were 5.1 ± 2.1 h (46%) (*p* = 0.29) and 2.2 ± 1.0 h (84%) (*p* = 0.34).

Women's satisfaction with delivery when asked before discharge from the hospital was comparable with VAS score 7.7 ± 2.0 after continuous support and 7.8 ± 2.1 after standard care (*p* = 0.61). The mean hospital stay was 2 days after vaginal delivery and 3 days after emergency CS.

Maternal cortisol levels are shown in Table 2. Women in the continuous support group tended to have lower cortisol levels during all stages of active labor compared to women with standard care – during the first stage 1,199 ± 482 nmol/L compared to 1,363 ± 503 nmol/L (*p* = 0.28), during the second stage 1,301 ± 614 nmol/L compared to 1,378 ± 682 nmol/L (*p* = 0.72), and during the third stage 1,273 ± 413 nmol/L compared to 1,426 ± 533 nmol/L (*p* = 0.30). Low cortisol during the first stage of active labor was correlated to shorter duration of the first stage (*p* = 0.02) but not to duration of the second stage (*p* = 0.59). Likewise, low cortisol during the second stage was correlated to shorter duration of the first stage (*p* = 0.04) but not to duration of the second stage (*p* = 0.93). Maternal cortisol during the third stage was not correlated to the length of the first (*p* = 0.17) or second (*p* = 0.68) stages of active labor. Serum samples were not obtained from all women due to practical reasons.

**Table 2:** Maternal serum cortisol.

**Variable**	**Continuous support** ***n* = 30**	**Standard care** ***n* = 29**	***p*-value**
**S-cortisol nmol/L**, mean ± SD			
First stage of active labor, *n* (%)	*n* = 27 (90) 1199 ± 482	*n* = 22 (76) 1363 ± 503	0.28^a^
– Correlation to duration of 1st stage			0.02^b^
– Correlation to duration of 2nd stage			0.59^b^
Second stage of labor, *n* (%)	*n* = 23 (77) 1301 ± 614	*n* = 14 (48) 1378 ± 682	0.72^a^
– Correlation to duration of 1^st^ stage			0.04^b^
– Correlation to duration of 2nd stage			0.93^b^
Third stage of labor, *n* (%)	*n* = 24 (80) 1273 ± 413	*n* = 16 (55) 1426 ± 533	0.30^a^
– Correlation to duration of 1st stage			0.17^b^
– Correlation to duration of 2nd stage			0.68^b^

a Statistical methods Mann Whithey U-test and

b *General Linear Model*.

Neonatal data are shown in Table 3. The BW (mean ± SD) was comparable 3,617 ± 567 g between the continuous support group and 3,447 ± 489 g the standard care group (*p* = 0.51). There were no signs of neonatal asphyxia expressed as an Apgar score <7 at 5 min or a BE(a) < -10 mmol/L. The mean Apgar score at 5 min was comparable 9.8 ± 0.5 in the continuous support group and 9.8 ± 0.4 in the standard care group (*p* = 0.85). None of the neonates in either of the groups was admitted to NICU care. There were no neonatal deaths.

**Table 3:** Neonatal data.

**Variable**	**Continuous support** ***n* = 30**	**Standard care** ***n* = 29**	***p*-value**
Birth weight, g (mean ± SD)	3,617 ± 567	3,424 ± 495	0.51
Apgar score at 5 min, points (mean ± SD)	9.8 ± 0.5	9.8 ± 0.4	0.85
Apgar <7 at 5 min, *n* (%)	0	0	
BE(a) < -10 mmol/L, *n* (%)	0	0	
NICU admission, *n* (%)	0	0	

*Statistical method Mann Whitney U-test*.

*BE(a), base excess umbilical artery; NICU, Neonatal Intensive Care Unit*.

## Discussion

Although labor support by a midwife is described as early as during the 13th century BC in The Book of Exodus (*Shemot, Heb*.), where midwives are mentioned by their names Shiphrah and Puah (The Hebrew Bible, 2019), further understanding about the impact of labor support is still warranted in today's obstetric practice (Kennell et al., 1991; Bohren et al., 2017; World Health Organization, 2018; Uvnas-Moberg et al., 2019; Olza et al., 2020). Whether continuous labor support promotes labor progress and spontaneous delivery is of high clinical importance, since most instrumental deliveriers and emergency CS are carried out because of prolonged labor. These interventions increase the risk of complications such as obstetric bleeding, infection, perineal lacerations and a negative birth experience (Simic et al., 2017; da Silva Charvalho et al., 2019). The obstetric care and teamwork which involves obstetricians, midwives and nurses is dynamic and complex. Interprofessional collaboration and continuous, respectful communication with the laboring woman and the couple is crucial for high-guality medical safety and satisfaction with delivery. Different regional, hospital and professional cultures can constitute barriers and counteract constructive interprofessional collaboration (Romijn et al., 2018).

In this randomized controlled study, we have investigated the duration of active labor, delivery mode, need of labor analgesia, satisfaction with delivery, and cortisol levels among primiparous women who received continuous support as compared to labor support according to standard clinical routines. Furthermore, we have investigated the neonatal morbidity of their infants. The main findings were, that continuous support was followed by shorter active labor than was labor support according to standard clinical routines. Continuous support was followed by a high rate of vaginal delivery 97% compared to 86%, and a low emergency CS rate 3% compared to 14%. Women in the continuous support group tended to receive EDA more seldom and fewer EDA doses, and they received combined modes for labor analgesia more often. Women's satisfaction with delivery did not differ between the groups when asked before discharge from the hospital. Maternal cortisol in they continuous support group tended to be lower during all stages of active labor and low cortisol was correlated to shorter active labor. No cases of neonatal distress were observed, none of the neonates was admitted to NICU care, and there were no neonatal deaths.

The duration of active labor among primiparous women with continuous labor support was shorter not only when compared to women with standard care in the present study, but also when compared to the duration of active labor among primiparous women with singleton pregnancy and spontaneous labor onset in a large contemporary study (Zhang et al., 2010). Based on the present findings of a tendency toward lower cortisol among women with continuous support and the correlation between low maternal cortisol and shorter active labor, taken together with previous reports showing that cortisol represents stress during delivery (Miller et al., 2019), we concluded that women with continuous support had a lower stress level which promoted labor progress. The present results were in agreement with previous studies reporting that high emotional stress and anxiety are related to longer duration of labor (Lederman et al., 1985; Alehagen et al., 2005; Hishikawa et al., 2019; Miller et al., 2019; Uvnas-Moberg et al., 2019; Olza et al., 2020).

Women with continuous support tended to use EDA more seldom and fewer EDA doses, and they received more combined modes of labor analgesia – inhalation of a mixture of nitrous oxide and oxygen (50/50%), acupuncture and /or pudendus blockage – than women with standard care, which could have contributed to labor progress. Combined methods for labor analgesia were positively correlated to shorter labor in the present study. Reports of longer labor and lower circulating endogenous oxytocin levels among women with EDA compared to women without EDA attribute these effects to a block of parasympathetic fiber mediated oxytocin release, known as the Ferguson reflex (Flint et al., 1978; Rahm et al., 2002; Anim-Somuah et al., 2018). Some authors (Miller et al., 2019) but not all (Alehagen et al., 2005) report lower cortisol levels in laboring women with EDA compared to women without EDA.

The theoretical explanations for the mechanisms behind labor support on childbirth outcomes hypothesize that labor support enhances labor physiology and mothers' feelings of control and competence, and by reducing fear, anxiety, pain, and reliance of medical interventions (Kennell et al., 1991; Bohren et al., 2017; World Health Organization, 2018; Uvnas-Moberg et al., 2019; Olza et al., 2020). It is well-documented, that components of labor support such as human presence, psychological support and physical touch facilitate endogenous release of the anti-stress hormone oxytocin, and that oxytocin counteracts anxiety, fear, the experience of pain and decreases levels of the stress hormone cortisol (Uvnas-Moberg et al., 2019; Olza et al., 2020). Furthermore, maternal oxytocin release is not only positively correlated to labor progress and reduced stress and anxiety, but also to the onset of breastfeeding (Nissen et al., 1996). Consequently, maternal oxytocin levels during breastfeeding are inversely correlated to cortisol levels (Handlin et al., 2009). Kennell and Klaus et al. have shown in their pioneering studies, that the presence not only of a supportive person during labor but also the presence of an observer, who stayed in the labor room at some distance from the mother during the entire labor had significant effect on obstetrical outcomes even though she did not speak with the mother. The authors point out, that the observer effect may be solely her presence in the labor room and its positive effects on the laboring woman and on the staff (Kennell et al., 1991).

The CS rate 3% among women with continuous support was lower not only when compared to the CS rate 14% among women with standard care in the present study, but also when compared to the CS rate 10% in primiparous women with spontaneous labor onset (Robson Group 1) at our hospital during the years studied 2015–17, and when compared to the CS rate 8% among primiparous women with spontaneous labor onset (Robson Group 1) in Sweden in 2015–17 (The Swedish Pregnancy Register, 2020). Maternal age and BMI might have been confounders regarding labor outcome since high maternal age and high BMI increase the risk of CS (Weiss et al., 2004; Londero et al., 2019). Nevertheless, maternal age and BMI in the present study were comparable to the mean age 29 years and mean BMI 24 among primiparous women in Sweden in 2015–17 (The Swedish Pregnancy Register, 2020). The present results were in accordance with the results by Kennell et al. (1991), and with a recent study on term pregnant primparous and parous women with spontaneous labor onset, where supportive care by a doula during childbirth is followed by a high rate of vaginal delivery 94%, compared to 92% with acupuncture and 60% with standard care (Akbarzadeh et al., 2014). Previous studies have shown, that fear of vaginal delivery among primiparous women is associated to a higher rate of planned CS in 19% compared to 3% in a reference group of women not fearing vaginal delivery (Sydsjö et al., 2014), and that most planned CS are performed because of fear of vaginal delivery and non-medical reasons (da Silva Charvalho et al., 2019).

Surprisingly, the rate of documented presence by a midwife or a nurse in the labor room did not differ between the groups. Even though the quality of labor support was not monitored here, we observed, that women in the continuous support group received longer periods of labor support whereas women in the standard care group received more fragmented support in shorter periods of 5–15 min according to standard clinical routines. It is possible, that the mere participation of women in the present study increased the focus on labor support and thereby the presence by a midwife or a nurse in both groups according to the Hawthorne effect, which describes that individuals may modify an aspect of their behavior when they are aware of being observed (McCambridge et al., 2014).

Strengths of this study were its randomized controlled design, the inclusion of primiparous women without intercurrent diseases or pregnancy complications only, and that all data were collected from original electronic obstetric records. Limitations were that midwives and nurses did not get any special training before start of the study and that continuous support was not provided according to standardized criteria. Since the power analysis was based on the primary outcome duration of active labor, the results regarding the secondary outcome rate of emergency CS may be biased. Also, the acute stress hormones catecholamines and the anti-stress hormone oxytocin were not monitored here.

Future studies on the impact of continuous labor support on childbirth outcomes, and on longterm effects such as bonding between the mother and infant, onset of breast-feeding and postpartum health are encouraged. Continuous support according to standardized criteria after special staff education, and with standardized protocols to monitor anxiety, fear and pain would add valuable information to this field.

In conclusion, the results of this randomized controlled study showed, that primiparous women who received continuous labor support by a midwife or a nurse had shorter active labor than primiparous women who received support according to standard clinical routines. Continuous support was followed by a high rate of vaginal delivery and a low rate of emergency CS. Maternal cortisol in the continuous support group tended to be lower during all stages of active labor reflecting a lower stress level and low maternal cortisol was correlated to shorter active labor. Based on these results, we recommend continuous labor support for all primiparous women during active labor.

## Data Availability Statement

The raw data supporting the conclusions of this article will be made available by the authors, without undue reservation.

## Ethics Statement

Ethics approval was obtained from the Regional Ethics Board for Medical Sciences in Stockholm October 9, 2013, Ref No. 2013/1079-31/3. The patients/participants provided their written informed consent to participate in this study.

## Author Contributions

PC, OB, and YS: collected data. YS, TV, and MP: analyzed the data and wrote the manuscript. The manuscript was read and approved by all authors.

## Conflict of Interest

The authors declare that the research was conducted in the absence of any commercial or financial relationships that could be construed as a potential conflict of interest.
